# A Multi-Agent Deep Reinforcement Learning Approach for Enhancement of COVID-19 CT Image Segmentation

**DOI:** 10.3390/jpm12020309

**Published:** 2022-02-18

**Authors:** Hanane Allioui, Mazin Abed Mohammed, Narjes Benameur, Belal Al-Khateeb, Karrar Hameed Abdulkareem, Begonya Garcia-Zapirain, Robertas Damaševičius, Rytis Maskeliūnas

**Affiliations:** 1 Computer Sciences Department, Faculty of Sciences Semlalia, Cadi Ayyad University, Marrakech 40000, Morocco; hananeallioui@gmail.com; 2 Computer Science Department, College of Computer Science and Information Technology, University of Anbar, Ramadi 31001, Iraq; mazinalshujeary@uoanbar.edu.iq (M.A.M.); belal-alkhateeb@uoanbar.edu.iq (B.A.-K.); 3 Laboratory of Biophysics and Medical Technology, Higher Institute of Medical Technologies of Tunis, University of Tunis El Manar, Tunis 1006, Tunisia; narjes.benameur@yahoo.fr; 4 College of Agriculture, Al-Muthanna University, Samawah 66001, Iraq; khak9784@mu.edu.iq; 5 eVIDA Laboratory, University of Deusto, 48007 Bilbao, Spain; mbgarciazapi@deusto.es; 6 Faculty of Informatics, Kaunas University of Technology, 51368 Kaunas, Lithuania; rytis.maskeliunas@ktu.lt

**Keywords:** multi-agent reinforcement learning, COVID-19 segmentation, CT image, mask extraction, semantic segmentation

## Abstract

Currently, most mask extraction techniques are based on convolutional neural networks (CNNs). However, there are still numerous problems that mask extraction techniques need to solve. Thus, the most advanced methods to deploy artificial intelligence (AI) techniques are necessary. The use of cooperative agents in mask extraction increases the efficiency of automatic image segmentation. Hence, we introduce a new mask extraction method that is based on multi-agent deep reinforcement learning (DRL) to minimize the long-term manual mask extraction and to enhance medical image segmentation frameworks. A DRL-based method is introduced to deal with mask extraction issues. This new method utilizes a modified version of the Deep Q-Network to enable the mask detector to select masks from the image studied. Based on COVID-19 computed tomography (CT) images, we used DRL mask extraction-based techniques to extract visual features of COVID-19 infected areas and provide an accurate clinical diagnosis while optimizing the pathogenic diagnostic test and saving time. We collected CT images of different cases (normal chest CT, pneumonia, typical viral cases, and cases of COVID-19). Experimental validation achieved a precision of 97.12% with a Dice of 80.81%, a sensitivity of 79.97%, a specificity of 99.48%, a precision of 85.21%, an F1 score of 83.01%, a structural metric of 84.38%, and a mean absolute error of 0.86%. Additionally, the results of the visual segmentation clearly reflected the ground truth. The results reveal the proof of principle for using DRL to extract CT masks for an effective diagnosis of COVID-19.

## 1. Introduction

Human beings can easily recognize the visual world. However, it is quite difficult to interpret a computed tomography (CT) image. The goal is to be able to process CT features more precisely by avoiding coarse detection to fine prediction of each pixel while guaranteeing system performance regarding improving its adaptability by self-learning from previous experiences. Initially, texture analysis is an important operation for segmentation, classification, recognition, and detection of two-dimensional (2D) images. Although much important work has been done in the 2D context, techniques addressing CT volume, such as CT mask extraction, were not thoroughly covered. Up until now, the mask image representing the ground truth was usually arbitrarily selected from a series of analyses performed by experts.

The available budget for manual mask extraction plays a crucial role in the segmentation of CT images. Although numerous segmentation techniques automatically segment medical images for clinical diagnosis [1,2,3], when an unexpected and unprecedented pandemic occurs, as in the case of COVID-19, the training datasets and annotations are almost nonexistent, making the application of supervised artificial intelligence (AI) methods complicated [4,5]. In addition, experts face an unpredicted burden that makes the cost of manual mask extraction from lung CT images much higher. Consequently, reducing human effort in COVID-19 CT mask extraction can optimize time and CT segmentation costs while providing highly relevant results. For that, a multi-agent system (MAS) is qualified to learn mask extraction policies, in a deep reinforcement learning context, to reach optimal semantic segmentation of COVID-19 CT images.

In semantic segmentation, each pixel of an object belonging to a particular class receives the same label/color value [6,7]. In contrast, in instance segmentation, each pixel of each object in a class receives a separate label/color value. Mask image extraction (masking) is then part of instance segmentation. We can automatically compute pixel-wise masks for objects in the CT image, allowing us to segment the foreground from the background. Current medical datasets, made available to the public, are quite rare or even nonexistent in some specialties [8]. This raises several important problems:

- Manual extraction and quality control take hours to process an image.
- Classical or manual labeling, at the pixel level, consumes a lot of time.
- The class discrepancy in the image datasets varies greatly, which may result in unwanted influences in the learned methods.

This appears when extracting images from masks to create the semantic segmentation of a new dataset. These problems could be avoided by precisely selecting regions that refer to certain masks [9]. Medical image segmentation and object detection distinguish among the various objects that make up the CT image [10]. Masking is one of the techniques used to draw frames for the detected object and to determine if the pixels in the frame belong to such an object [11]. This is used to detect the object and identify its boundaries [12]. The masking process is used to extract features, classify the bounding boxes, and quantify each region of the image to facilitate medical diagnosis [13]. For example, a simple object detection frame in a medical image may not work well because the object is simply detected, and then a fixed shape is drawn around it. This is a risky proposition in a real medical scenario. Imagine that there is a small object near the surrounded object, and we draw a rectangular box around the large object because it has more intensity. The observer of the result may not understand the arrangement of the objects correctly and may even ignore areas that will have a significant impact on the patient′s case.

Instead, we need a technique that can detect the exact shape of any object in the image so that the segmentation system can detect diseases. An advanced mask extraction method is developed based on the reinforcement learning framework to perform all source classification tasks in a novel learning framework. Thus, we propose a novel real-time segmentation system utilizing our key multi-agent system Reinforcement learning in a cooperative way to detect and generate the mask of an object in an image, extract the detected objects (all or part of them) from different CT medical images, and then collect them together to form a solid base for the training phase. CT images are often degraded by the mask image. The objective of this work is to extract optimal image masks using a multi-agent reinforcement learning (MARL) process.

MAS is a set of agents that share a common environment through sensors, on which they act through actuators [14]. One of the main advantages of fully cooperative agents is that they aim at achieving the same goal. To this end, we have chosen exclusively to opt for cooperative multi-agent systems along with multi-agent reinforcement learning (MARL). Normally, in MASs, the environment is so complex that a priori design of good agent behavior is not practical. Thus, learning is most needed in these systems and, because of its simplicity and convergence properties, reinforcement learning (RL) provides a good basis for multiagent learning. Consequently, RL methods are used in the MAS framework, which is called MARL [15]. Because of its decentralized structure, the MARL architecture can provide several advantages, since it allows us to break down a globally complex problem into more tractable subproblems. A major advantage is that the action space to be searched for each agent can be organized so that only related action spaces are included. In addition to the computational advantages, the size of the action space to be searched for each agent is reduced, which can result in faster learning. Furthermore, the ability to share experience between agents and the robustness properties of MARL make it an active area of study [15].

To achieve optimal medical semantic segmentation efficiency, we attempted to learn mask extraction task policies. Therefore, an advanced solution is presented based on MAS reinforcement learning. Such an approach is suitable for the current semantic segmentation needs. Thus, our contributions can be summarized in the following points:

- Introducing an effective system to enhance semantic segmentation by modifying the traditional Deep-Q-Network to learn superior mask extraction compared to the most advanced methods.
- Proposing a multi-agent reinforcement learning (MARL) framework to extract COVID-19 masks automatically to improve the efforts of CT experts and enhance the potential of lung CT segmentation. We selected learning in the Markov decision process (MDP) to find optimal approaches in mask extraction for CT experts.
- Reducing the waiting time for experts to obtain masks manually, by introducing an alternative automatic approach.

Section 2 reviews previous studies that discussed RL and medical semantic segmentation. Section 3 discusses the semantic segmentation environment. Section 4 covers the results and discusses the foundation of the current study. Section 5 states the conclusion.

## 2. Related Works

Approaches in medical image segmentation can generally be categorized into unsupervised or semi-supervised methods. These approaches are either trained on a subset of the target dataset or pre-trained on datasets that are comparable to the target dataset. Recently, several studies have been based on deep learning [16,17,18]. However, in a medical environment, such models may become semantically biased, resulting in severe performance loss. Labeling images, especially medical images, requires time and high attention to diagnose diseases. Practically, the remaining medical image segmentation techniques are based on neural networks. The last few years have brought a rapid advance in deep learning techniques for semantic segmentation with promising results. The first milestone of deep learning was the FCN by Long et al. [19]. In numerous well-known architectures, FCN was used to cast the fully convolutional layers: e.g., AlexNet [20], VGG-16 [16], GoogleLeNet [17], ResNet [18]. FCN provides a hopping architecture that allows the fine layers of the network to visualize and analyze information from the coarse layers. This increases the capacity of the model to handle a global context while improving the quality of the semantic segmentation. However, with an increasing number of layers, the receptive field of FCN filters increases linearly. This influences local predictions when integrating global knowledge [21]. Hence, later researchers improved the capacity of their image models to process the global context with different approaches.

Several works have studied the division of an image into semantically meaningful regions as well as the clustering of the found regions. During the training phase [22], processed the image composed of noises by limiting the number of annotated samples (image-level annotation, not pixel-level). Label consistency and inter-label correlation were examined by training CRF from the weakly labeled image [23]. Multiple-task and multiple-instance learning were employed by [24] to segment semantically the images weakly annotated using geometric context estimation. Xu et al. [25] deployed weak annotation to reduce the label complexity; this model was needed to decrease labeling cost. A neural network and iterative graphical optimization were combined by Rajchl et al. [26] to approximate pixel-wise object segmentation. FCN was widely used independently or cooperatively with other methods [27,28,29,30,31]. In some of these methods, FCNs report some deficit for local dependency coverage, so the implementation of new approaches is still a strong point for developing semantic segmentation models.

In the context of segmentation of infections and lesions, very promising deep learning systems have been suggested for medical image analysis [32,33,34,35,36,37]. Mostly, this work is dedicated to segmenting the lungs and the classification of regions of infection to support clinical evaluation and diagnosis [38]. In the case of COVID-19, segmentation allows delineating regions of interest in lung images for further evaluation. Wang et al. suggested a weakly supervised deep learning software system that uses CT images for the detection of COVID-19 [39]. Gozes et al. presented a system that studies 2D slices to analyze 3D volumes [40]. Wang et al. presented a rapid diagnostic system for COVID-19, based on segmentation for the localization of lung lesions and then classification to determine the lesion analogy [41]. Li et al. introduced a COVNet to obtain visual information from volumetric chest CT scans to distinguish community-acquired pneumonia (CAP) from COVID-19 [42]. Chen et al. proposed the use of the U-Net++ network for extraction of COVID-19 affected areas and detection of suspicious lesions on CT images [43]. Akram et al. [44], to extract the relevant features, used discrete wavelet transform and extended segmentation-based fractal texture analysis methods followed by an entropy-controlled genetic algorithm to select the best features from each feature type, which were then combined serially.

Khan et al. [45] began with a contrast enhancement using a top-hat and Wiener filter combination. Two pretrained deep learning models (AlexNet and VGG16) were fine-tuned based on the target classes (COVID-19 and healthy). A parallel fusion approach—parallel positive correlation—was used to extract and fuse features. The entropy-controlled firefly optimization method was used to select optimal features. Machine learning classifiers such as the multiclass support vector machine (MC-SVM) were used to classify the selected features and achieved 98% accuracy using the Radiopaedia database. Rehman et al. [46] proposed a framework for the detection of 15 different types of chest diseases, including COVID-19, using a chest X-ray modality. A two-way classification was performed. The first step used CNN architecture with a soft-max classifier. Second, transfer learning was used to extract deep features from the CNN′s fully connected layer. Deep features were fed into traditional machine learning (ML) classification methods, which improved the accuracy of COVID-19 detection and increased predictability for other chest diseases.

Reinforcement learning approaches have recently attracted more interest due to the effective union of RL and deep networks by Mnih et al. [47]. RL has been used extensively in numerous computer vision problems, e.g., video content summarization [48], tracking [49], object localization [50], and object segmentation [51]. Previously, numerous papers highlighted the importance of optimizing human efforts through interactive or automatic annotation of images or videos studied using RL. For example, the GrabCut algorithm was improved by generating seed points with a Deep-Q-Network (DQN) agent [52]. Acuna et al. [53] evaluated their approach in a human-in-the-loop structure. Rudovic et al. [54] integrated RL into an active learning context, so that the agent could decide whether the labeling is required from the user or not.

Although each of these methods has shown strong performance, they have a lower likelihood of scaling new high-level semantic information in training data. Inspired by the mentioned RL works, our approach utilizes DRL to straighten CT segmentation by an advanced mask extraction approach. We note that the use of MAS is ideal for extracting regions of interest to properly form the mask base for segmentation, since it provides a partitioning of the lung CT image at an arbitrary level of granularity.

## 3. The Proposed Semantic Segmentation Environment

**Figure 1:**
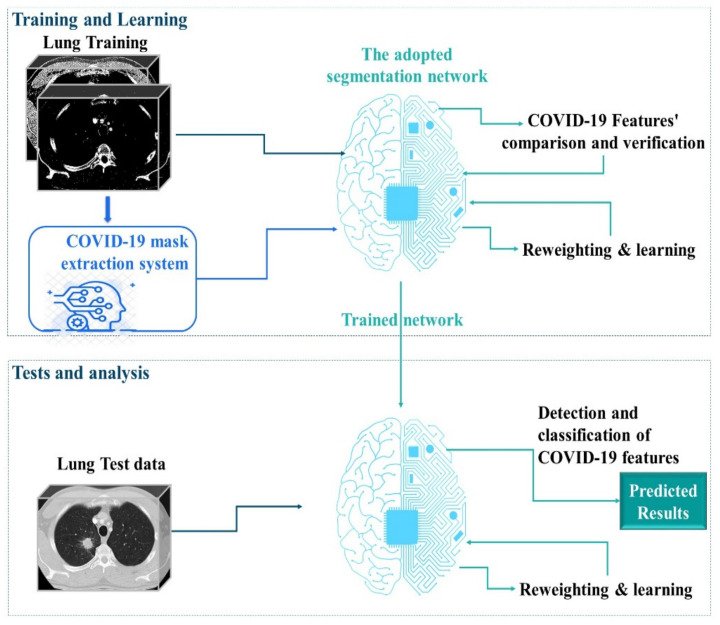
Overview of the proposed method: 1. A complete mask extraction is processed using the automatic COVID-19 mask extraction system. 2. The adopted segmentation network is trained using the obtained masks. 3. The segmentation network can segment CT images and provide strong predictions. Coronavirus disease, COVID-19.

In our study, we used MARL to automatically extract COVID-19 masks to optimize the efforts of CT experts and improve the potential of lung CT segmentation. We opted for learning in the Markov decision process (MDP) to develop effective mask extraction techniques for CT specialists. We employed an advanced approach to segment lung CT images to reduce human efforts. In the process described in Figure 1, the mask extraction system is responsible for the automatic extraction of the COVID-19 mask from CT images for a complete and optimized semantic segmentation. The RL system is trained to select optimal COVID-19 masks by estimating the magnitude of probable divergences between anticipated and image-derived characteristics. Masks can be considered as separators, allowing the transformation of raw data from 3D space to relevant observation units for image processing. In this phase of our research, we have extended a multi-agent reinforcement learning (MARL) approach to handling mask image extraction. For that, the following subsections describe the RL architecture for mask extraction, as well as detail its important functionalities (the Markov decision process and the mask detector).

### 3.1. RL Architecture for Mask Extraction

**Figure 2:**
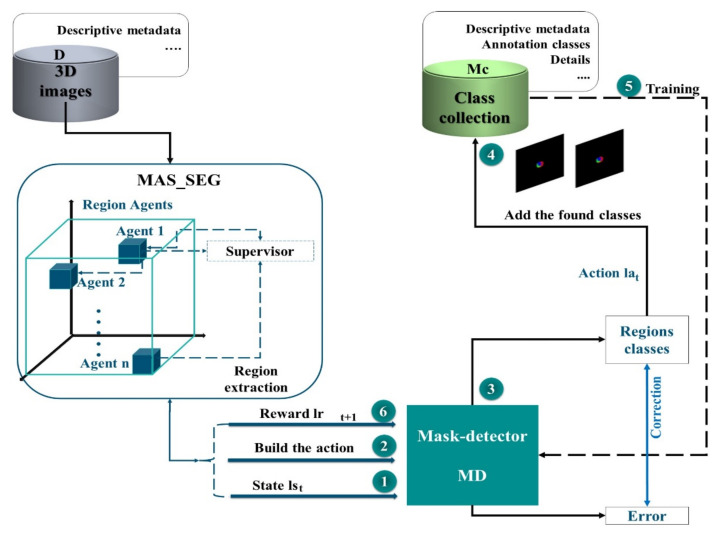
Architecture for Tree-dimensional mask extraction using Reinforcement learning. Three-dimensional, (3D).

An end-to-end method was suggested through the RL strategy to establish a learning policy that can discover the most informative regions in an image and extract the corresponding mask image. In this way, the network can achieve high-quality results. This architecture aims at producing masks that allow assigning masks to each voxel as a ground truth label. The algorithm may emphasize the most important portions of the images by picking selected areas rather than whole images. In addition, learning approaches that use an adapted DQN are explored to extract mask images more efficiently (Figure 2). First, we train the mask detector, selecting regions from the segmentation result of the MAS agents, to enhance the performance of the network. The process represented in this architecture is performed repetitively until a certain budget *B* of class samples is obtained.

During each iteration t, once the feature map of the MAS system is obtained, it is passed to the mask detector (MD) to be scanned. The MD is a network that scans thousands of masks, representing overlapping areas with different sizes and aspect ratios, so that two outputs can be generated for each mask and associated mask class. After processing, these masks will be added to the collection of classes ($M_{c}$), which is subsequently used to train our network. Intersection-over-Union (IoU), which is a typical semantic segmentation measurement, is used to assess performance. In this architecture, we use MAS and deep learning techniques to overcome the potential errors of traditional methods for image mask extraction.

### 3.2. Structural Properties of MAS_SEG

Being autonomous and independent learners, the MAS agents interact with the image in an organized framework, which we named MAS_SEG. The complexity of the segmentation algorithm adopted in MAS_SEG is relative to the number of states and actions. Otherwise, while learning in the 3D image as a fully shared state-action space, MAS agents must be informed of other agents’ decisions (i.e., actions or both actions and rewards) and keep track of the action values for each of them; when the shared information contains both actions and rewards, the complexity increases.

**Figure 3:**
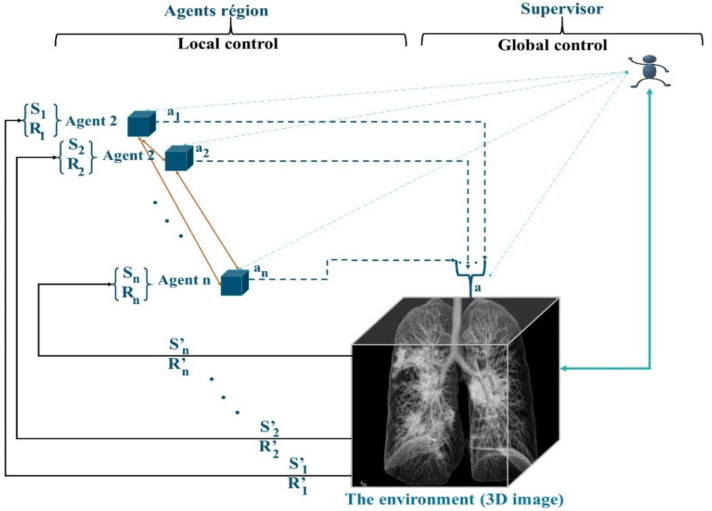
RL Structure of MAS_SEG.

The preparation of the regions is performed by MAS_SEG, and this system is based on two techniques, the principles of RL learning and a nonstationary policy to produce a cooperative segmentation. Often, RL methods guarantee the learning of the optimal behavior of an agent based on a reward obtained from its environment. Most of the works in RL learning consider that the agent has only one goal to maximize. However, in the case of agents in MAS_SEG, each agent must perform many sequential decision tasks, which reveals multiple conflicting goals. For example, in the 3D segmentation task, merging regions is a competitive task, which requires reducing conflicts of interest and increasing the detection of regions satisfying similarity criteria, achieving optimal system stability, and considering interaction time and the lightness of merging in a cooperative and efficient context. To this end, the agents in MAS_SEG illustrated in Figure 3 admit a policy that best fits a predefined trade-off between objectives.

Agents participate in image segmentation by region merging, with the simple difference that agents are in the image by adopting a supervoxel algorithm to determine the starting regions instead of the superpixel. Agents maneuver according to a negotiation mechanism using game theory techniques to properly identify concerns about the merging of regions. In addition to fusion tasks, agents in MAS_SEG learn to act in the 3D image by being guided by the rewards received from the environment. The objective is to improve the mask identification process by increasing the learning speed and improving the learned policy.

### 3.3. The MAS Q-Learning Algorithm and the ε-Greedy Strategy

The suggested QL-MAS protocol is described, where the *Q* learning technique is combined with MAS_SEG performing region fusion segmentation for mask image preparation. The action $A_{i}$ affects the environment adjacent to the actions of other agents. The combination of such actions formulates the joint action $a_{k}$. However, an agent may be inactive among such actions, and does not trigger its *Q*-value update cycle. The environment reveals the new state ${Si}^{\prime }$ and rewards ${Ri}^{\prime }$ to the agent Agi at the end of the episode. The action-value function *Q*-value, Q, is:

(1) $$Qc+1\;\left(Sc,\;Ac\right)=\left(1\;-\;\alpha c\;\left(Sc,\;Ac\right)\right).\;Qc\;\left(Sc,\;Ac\right)+\alpha c\;\left(Sc,\;Ac\right)\;\cdot \;\left[Rc+1+\gamma \;\cdot \;max\;\left(Qc\;\left({Si}^{\prime },\;{Ri}^{\prime }\right)\right)\right]$$

with:

- $S_{c}$, $A_{c}$, $R_{c}$, and $Q_{c}$ represent respectively the state, action, reward, and *Q*-value on the *C*th episode.
- $\alpha c\;\in \;\left[0,\;1\right]$ is the learning factor of the *C*th episode.
- $\gamma \;\in \;\left[0,\;1\right]$ is the discount factor.
- α indicates the weight the system gives to new rewards by discounting the long-term Q value.
- γ is the factor that reduces the contribution of max, which is the maximum *Q*-value expected in the new state.

In our MAS_SEG, an episode is defined by a fusion within the image to be segmented. As a result, the state-action-state transition and the attainment of the reward occur only after the process of detecting areas has concluded.

During an image region discovery, the same action is performed. Actions are selected according to the ε-greedy strategy, depending on the exploration factor $\varepsilon \;\in \;\left[0,\;1\right]$. If ε≫0, the actions are taken arbitrarily from the actions’ set U ($a\;\in \;U_{\left(a_{min},a_{max}\right)}$) with high probability, to learn how the environment reacts to the different decisions. Otherwise, when ε≪1, the system exploits the knowledge gained by choosing the actions that yielded the maximum action values. Actions are chosen by comparing a random value *x* ∈ *U* [0, 1] with the parameter ε, which can vary over time:

(2) $$a=\left\{\begin{array}{c} U_{\left(a_{min},a_{max}\right)}\;\;\;\;\;\;\;\;\;\;\;\;\;\;\;\;if\;x\leq \varepsilon \\ \arg maxQ_{ep}\left(s_{ep},a\right)\;if\;x>\varepsilon \end{array}\right.$$

With: *Q_ep_*, *S_ep_* which respectively represent the *Q*-value and the state of the current episode ‘*ep*’.

The actions represent the available segmentation (region merge) results. The state is a combination of average merges and attempts to evaluate neighboring regions. The state then describes the behavior and policy of an agent linkage, providing an evaluation of the region merge. The state is expressed as:

(3) $$S_{C}=\|NbF_{C}\|+\|NbPf_{C}\|.\left(N_{f}+1\right),$$

with $N_{f}$ representing the maximum merges and $NbF_{C}$ and $NbPf_{C}$ representing the number of merges and merge proposals, respectively, at the C-th episode. Each merger is attempted for a maximum number of $NbPf_{C}$, max $NbPf_{C}$, which is reset to zero after the proposal is sent.

Rewards are a combination of the Pap, which is defined as the proposal approval. It is a percentage of agents who successfully negotiate a proposed merger of a region from the processed region map, quantified linearly on a merger number scale $N_{f}$ and number of final regions $N_{rf}$. A higher reward indicates a successful Pap and lower number of final regions, while a lower reward indicates failed Pap and a higher number of final regions. The reward calculation function is expressed as:

(4) $$R_{C}=\alpha .\left[N_{rf}\left(Pap_{C}^{q}-1\right)+\left(N_{rf}-F_{C}\right)-\frac{m}{2}\right]$$

where *α* is the size factor between two consecutive merge regions, *F_C_* is the number of merges (fusion) at the *C*th episode, m is the reward quantization factor and is expressed as $m=N_{f}-N_{rf}$. $Pap_{C}^{q}$ is the agent approval with a *Q*-matrix containing the *q*-values of the state-action combinations at the *C*th episode. The *Q* values are initially set to zero.

### 3.4. Role of the Supervisor Agent

Although the application of the reward difference strategy can change the equilibrium achieved by the agents, alternative policies affecting the agents might be learned. However, under this cooperative framework, the collection of alternative policies that may be learned and their relationships to one another remain stable. For MAS_SEG, the exploration of the agents, their cooperation, and the reinforcement learning mechanism are used to determine an extraction of homogeneous regions from the image. However, the map of discovered regions must be verified to ensure the reliability of the rest of the masking process.

The QL-MAS protocol is implemented in the customized MAS_SEG system with:

- Regional agents that cooperate.
- A supervisor agent, which incorporates a neural network to manage the interactions of the MAS agents and subsequently participates in the learning of the mask detector.

The reinforcement learning process in MAS_SEG is carried out according to the following QL-MAS algorithm (Algorithm 1).
**Algorithm 1.** QL_MAS  Initialize: The capacity Cap of the memory *M*, the values *Q*: ∀*r*, *a*|*Q* (*r*, *a*) = 0, The estimation weight
 LSTM-DQN *θ* = *θ*_0, the weight of the LSTM-DQN objectives *θ*′
 For episode = 1 → *ep* do # *ep* represents the number of episodes
  Fix the initial positions of the agents according to the map from the RAG
  For *i* = 1 -> Reg do # Reg is the number of regions
   #*N* is the number of agents *N* = Reg
   Implement in each Reg *i* an agent *A_i_*
  End for
  Do as long as *t* < Cap
   For *j* = 1 -> *N* do
    Calculation of the initial actions (2)
    Calculation of initial *Q*-value (1)
    Verification of the best adjacent neighbors satisfying the similarity criteria
    Negotiation to decide the optimal proposal
   End for
    Fusion
    Update of the map of the regions
    Update Reg
    *N* = Reg
   For *j* = 1 -> *N* do
    Calculation of the actions (2)
    Calculation of *Q*-value (1)
    Calculation of reward (4)
    Next state calculation (3)
    Save data *d* = {state(*t*), action(*t*), R(*t*), state(*t* + 1)} in memory *M*
    End for
   End Do
  Reset
 End for

The MAS_SEG system is implemented by the cooperation of MAS agents, which check the similarity criteria to perform the merging of regions to provide a final map of the segmented regions. However, it is essential to check these regions to indicate the return on investment. In MAS_SEG, each agent starts at a point belonging to a specific region. Then, it decides on which region of the image it will move, to perform the merging of the two regions. At each step, this agent has the information about its current region (size, similarity criteria, neighbors, etc.) and can select to go to another zone (move left, right, down, or up) or to stay in place. All agents are rewarded once they have fulfilled their selected actions. Agents must coordinate their actions to maximize the social welfare or overall utility of the system.

In this part of the system, we added the LSTM-Sharp model [55], which is an adaptable architecture to accelerate RL learning via long short-term memory (LSTM) and address the computational time challenges of agent interactions. In addition, we used the dynamics of the LSTM-Sharp computational engine to improve the adaptability of the learning. Through dynamic reconfiguration, this LSTM architecture adapts to the characteristics of MAS_SEG, including memory padding caused by MAS agent messages.

### 3.5. Region Generation Proposal

The generated feature maps are submitted to the MD, which scans the feature maps using a sliding window to locate the places where the object exists. Each obtained region is displayed on the image by a bounding rectangle (anchor). The MD produces the anchor category and correction data for the coordinates generating two outputs:

- The first output determines whether the anchor is in the foreground or background. If it is a foreground, it suggests there is an object in the rectangle.
- If the item is not properly located in the middle of the rectangle, the second output (error) is triggered to adjust the bounding box to best fit the detected object.

The mask detector then offers a series of boxes with details of their positions and sizes. If several boxes overlap, non-maximal suppression (NMS) is applied to obtain the box with the highest score in the foreground and move it to the next step. A size adjustment is made before the following stage to obtain a standard size. This accurately matches the retrieved characteristics with the original region proposal.

### 3.6. Markov Decision Process for Masking

Inspired by the research studies of [56,57], for mask extraction, we converted the active learning problem in a Markov decision process (MDP) formulation. For that, we modeled the mask detector as an RL agent, an enhanced deep Q network. Based on MAS_SEG information, this method enables the classification model to select policies based on the earlier RL experiences.

Our formulation differs from other approaches in the tasks we address, the definitions of states, actions and rewards, and the reinforcement learning algorithm we use to find the optimal policy. To detail how the presented process operates, we use four different data collections from the *Mc* mask class collection:

- The MD is trained using a subset of the TMc mask class collection to guarantee the active learning set for several episodes and learn a good acquisition function that maximizes performance with a budget of regions B.
- The MD network is tested on a separate Ev subset.
- To calculate the reward, we employ a different RMc subset.
- The state representation is built using the SMc set.

The MDP is characterized by the series of transitions $\left\{\left(ls_{t},\;la_{t},\;lr_{t}+1,ls_{t+1}:\right)\right\}$. For each state $\;ls_{t}\in S$, the MD is able to perform actions $la_{t}\in A$ to pick out the samples of $U_{d}$ to annotate.

The action $la_{t}={\left\{la_{t}^{k}\right\}}_{k=1}^{K}$, which consist of K sub-actions, is a function F exploiting the information of MAS_SEG. Each of the sub-actions requires a specific region to use a mask class. Next, it takes a reward $lr_{t}+1$ based on an increase in the average IoU per class when training the network.

As the MD′s states and actions do not depend on the specific architecture of MAS_SEG, they vary according to the operations performed by the MAS system. Thus, we intend to find a policy to select samples that maximize the performance of the global segmentation in the hybrid approach. We used a Deep-Q network (DQN) [47] and samples from an experiment buffer *ε* to train MD. Each episode ‘*ep*’ runs for a total of T steps. At each single iteration t, the subsequent steps are performed:

1. The state lst is calculated according to the value of the function *F* iteration t ‘Ft’ and  SMc.
2. A controlled action space is constructed with K group $G_{t}^{k}$ with N regions, sampled uniformly from the processed set D. For every single region within each cluster, we calculate its sub-actions representation $la_{t}^{k,n}$.
3. The MD, RL agent, selects *K* sub-actions ${\left\{la_{t}^{k}\right\}}_{k=1}^{K}$ using *ε*-greedy (greedy policy). Respectively, each single sub-action $la_{t}^{k}$ is defined as the selection of a region lrk (on N) to be processed from a set $G_{t}^{k}$.
4. A designation of the region masks is made, then the sets are updated: $Mc_{t+1}=Mc_{t}\cup {\left\{r1_{k}\right\}}_{k=1}^{K}$ (adding the new mask images in *Mc*) and $D_{t+1}=D_{t}/{\left\{r1_{k}\right\}}_{k=1}^{K}$ (removing these regions from set *D*).
5. The MD agent is trained through one iteration on the newly added regions ${\left\{lr_{t}^{k}\right\}}_{k=1}^{K}$.
6. RL receives the award rt+1 as the variation in performance between lft+1 and lft on RMc.

The end of each episode is reached when the budget B of the masks is reached, i.e., $\left|Mc\right|=B$. Once the episode ends, the MAS_SEG parameters are reset, and a new episode is launched. The MD is trained by simulating several episodes and updating its weights at each time step by sampling the transitions $\left\{\left(ls_{t},\;la_{t},\;lr_{t}+1,\;ls_{t}+1\right)\right\}$ from the experience replay buffer E.

### 3.7. State Representation

The global state of MAS_SEG is used and managed by the environment agent as an MDP state. It is quite complicated to integrate MAS_SEG in its entirety into a state representation. To reduce this complexity, the state space S is represented using a spared set SMc. A small portion of the training set is utilized to ensure that it has a meaningful representation of all classes.

**Figure 4:**
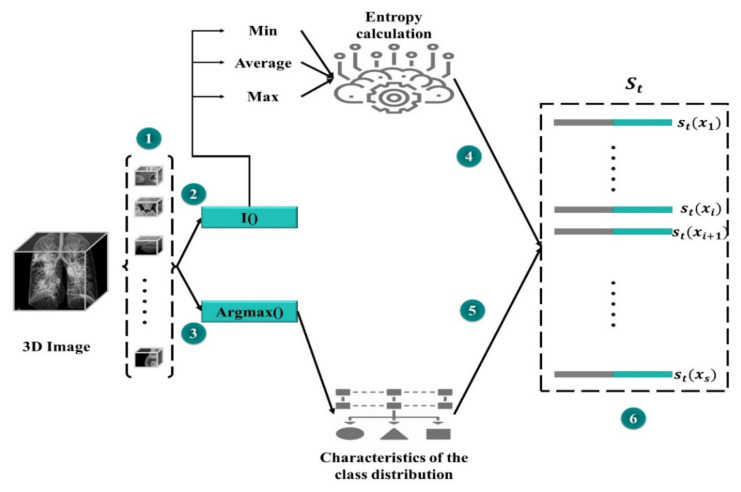
State representation of the mask detector.

The state set should have an equivalent class distribution into the training set. We believe this to be a typical subset of a specific dataset, and if there is any increase in segmentation efficiency on the SMc subset, the overall performance would be enhanced. The results of MAS_SEG on SMC are used to generate a universal representation of the lst state (step 1 in Figure 4).

A compact representation is essential to minimize high memory utilization owing to pixel-level predictions. SMc divides the data into segments, and compressed feature vectors are produced for each of them. Then, for each region, two feature sets are concatenated: one based on the feature vectors from MAS_SEG and the other one for the purpose of prediction uncertainty that is characterized by the Shannon entropy. The first group of features (i) is a (normalized) set used to extract projected number of pixels for each class. Furthermore, the predictor′s uncertainty with entropy is calculated as a function of the probability of the predicted classes. The entropy of each pixel position is calculated for each region to produce a map of spatial entropy. To wrap (compress) this representation, the entropy map is subjected to min, mean, and max pooling to produce a map of subsampled features. Another group of features (ii) is produced by flattening and concatenating the features of entropy.

### 3.8. Action Representation

**Figure 5:**
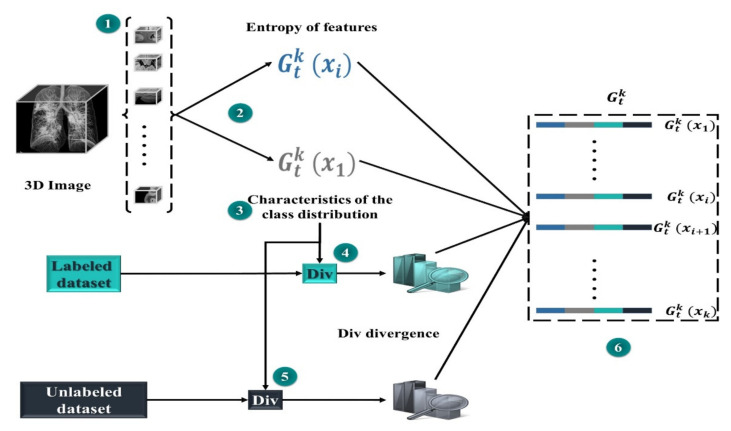
Representation of MD actions.

In our case, acting entails obtaining a mask. Owing to the large-scale nature of segmentation, processing the features of each region in the dataset at each step *t* would be extremely costly. Therefore, we estimate the set of regions of interest by sampling K groups $G_{t}^{k}$ from the collection of data to be masked *D*, each containing N uniformly sampled regions. We then perform the computation for the sub-action representation for each location $la_{t}^{k,n}$ (Step 2 of Figure 5).

Each sub-action $la_{t}^{k,n}\;$ is a series of distinct accomplished actions with class distribution and entropy features (as appeared in representation of the state), a similarity measure from one side between the region $x_{k}$ and class set Mc, and on the other side between region and set D. The idea is that MD will learn to build up a further class-balanced mask set while continuing to sample from set D. This could help in balancing the datasets of segmentation and improving whole performance. With each potential candidate region, x in a group $G_{t}^{k}$, we calculate the divergence (Div) between two sides, on the one hand, the class distributions in the region *x* for each prediction map (projected as the normalized number of predicted pixels in each class), on the other hand, the class distributions of marked and unmarked areas.

For the set of mask classes, we calculate a divergence (Div) score for the class distribution of each of the masks and region *x*. The greatest or sum of all of these (Div) could be utilized to summarize them. To acquire more specific information, we compute a normalized histogram of the Div divergence scores, which provides a distribution of similarities. For example, if we add up all scores, having half of the masks with a (Div) of zero and the other half with a *v*-value is like having all masks with (Div) of *v*/2. The final condition is more fascinating because it suggests there is no area that is the same as class distribution in *x*. The same approach is used with the dataset, producing a dissimilar distribution of Div discrepancies. Both are concatenated and appended to the representation of the activity. Figure 4 shows how each potential action is represented in a set.

### 3.9. Mask-Detector (MD)

**Figure 6:**
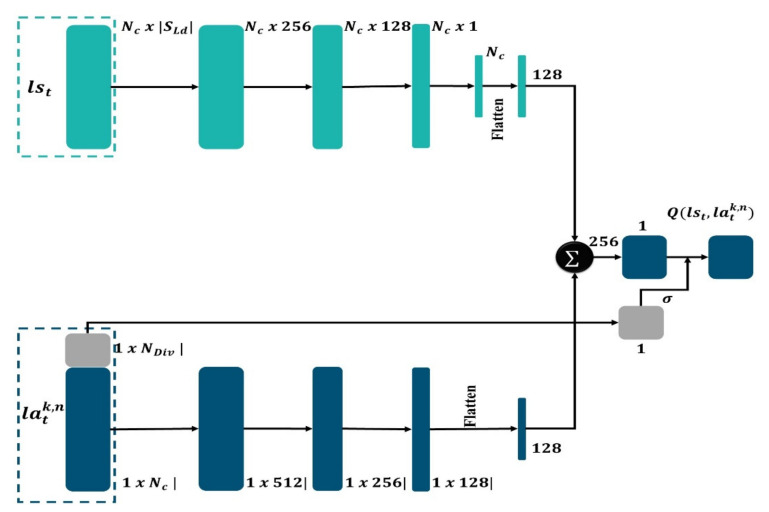
Schematic representation of MD architecture.

The mask detector is a network that has been upgraded to include a branch for producing segmentation masks. The MD network computes region proposals, then extracts features from each proposal and executes two concurrent processes. Object identification, categorization, and bounding box regression are all obtained. Following that, the other process develops high precision segmentation masks. Because MAS makes use of both high-resolution feature maps for precise localization, the agent operation is employed as a support in our work to produce advances in both accuracy and speed. While MAS_SEG is considered as the most advanced architecture in our approach, for each medical image, features are first extracted using the optimal agent strategy, and then MD computes the proposals to map spatial regions of interest of arbitrary size. Finally, MD (Figure 6) can estimate the object’s class, refine the bounding box position, and create segmentation masks all at the same time.

The MD is considered as a learning agent following an optimal policy to ensure efficient mask extraction. These policies link each state to a course of action that expands the total of projected future rewards. We count on a DQN, parameterized by *φ*, to obtain an ideal policy. We train our DQN with a TMc set of mask classes and then perform the rewards computing in the RMc subset. As previously stated, the MD technique picks K areas prior to proceeding to the subsequent state. We suppose that each region is chosen separately, as in the situation where a region is processed in parallel by a K number of MDs. On these terms, action comprises K independent sub-actions ${\left\{la_{t}^{k}\right\}}_{k=1}^{K}$, each having a partial action space, preventing the action space′s combinatorial explosion. To save time and avoid picking the same locations many times, we confine each single sub-action $la_{t}^{k}$ for selection to a region $x_{k}$ in $G_{t}^{k}$ described as:

(5) $$la_{t}^{k}=argmax\;Q\left(ls_{t,}la_{t}^{k,n};\varphi \right),$$

For each $k\;\in \;\left\{1,\;\ldots ,\;K\right\}$ action taken at time step t.

By way of loss optimization using the time difference error, the network is trained (TD) [58,59]. The loss is described as the expectation on the decomposed transitions *Tk* = {($ls_{t}$, $la_{t}^{k}$, $lr_{t+1}^{k}$, $ls_{t}$ + 1)}, which is obtained from the typical transitions $\left\{\left(ls_{t},\;la_{t},\;lr_{t+1},\;ls_{t}+1\right)\right\}$, by approximation of: $lr_{t+1}^{k}\approx lr_{t+1}$: $E_{T_{k}\sim \varepsilon }\left[{\left(o_{t}^{k}-Q\left(ls_{t},la_{t}^{k};\varphi \right)\right)}^{2}\right]$, where E is the rerun buffer of the experiment and $o_{t}^{k}$ is the objective of TMc for each sub-action $la_{t}^{k}$.

We employed a target array with weights and a dual DQN formulation to stabilize the training [60]. Action selection and assessment are separated; the action is chosen using the target network and assessed using the Annotator-DQN. Each sub-TMc action′s target is represented as follows:

(6) $$o_{t}^{k}=lr_{t+1}+\gamma Q\left(ls_{t+1},\;argmax\;Q\left(ls_{t+1,}la_{t+1}^{k,n},\varphi^{\prime }\right);\varphi \;\right),$$

where *γ* is a discount factor.

This formulation is correct if we consider the sub-actions to be independent of one another and conditional on the state. However, we noticed that at each phase, the expanded number of sub-actions K speeds up computation without compromising segmentation performance.

The MAS_SEG system, which is endowed with reinforcement learning capabilities, has the advantage of providing good region map extraction. The mask detector, as shown in Figure 6, consists of two distinct routes, the first one for the purpose of calculating state features and the other for calculating the action features, that will merge at the end. Each layer comprises batch normalization, ReLU activation, and a fully linked layer. The state path is made up of four layers, while the action path is made up of three layers, with a last layer that combines them to obtain overall features; they are linked by a sigmoid function that is under control by distributions of KL distance in representation of the action. At every single step into the loop of active learning, the weights are altered by random sample batches of 16 tuples from a 600-byte experience replay buffer.

The MD first selects a state representation and then selects an action representation for probable action. Nc represents the number of states and action features, whereas NDiv denotes the number of KL divergence distribution features. The features for the two representations are calculated individually, with layers consisting of batch normalization, fully connected layers, and ReLU activation. To adapt a last linear layer that records as a single scalar, the two-fold vectors of features are concatenated and flattened. The *Q* amounts are generated as the output score; therefore, the output is managed by a feature representation derived from the action representation′s distributions of the KL distance.

## 4. Results and Discussion

We will first offer information regarding the dataset collection as well as the assessment measures that were employed. Later, we evaluate our proposed method based on the DRL system using U-Net++ [43] as a semantic segmentation network to have a clear basis for comparison. Furthermore, we evaluated the DRL system to assess the quality of the masks learned by the reinforcement learning system. Finally, since the detection of masks in CT images is performed manually by experts, we compared our COVID-19 CT segmentation to the most advanced segmentation techniques.

### 4.1. Dataset Collection

Due to the recent appearance of COVID-19, none of the main archives had a substantial collection of COVID-19 labeled data, forcing us to rely on diverse image sources of normal, pneumonia, and COVID-19 cases. As a first step, different images were collected to obtain a very accurate semantic segmentation, which can facilitate the detection and classification of COVID-19 infections. We used four open datasets (detailed in Table 1):

- COVID-19-A: the public TCIA dataset [61] containing many CT lung scans (for non-COVID-19 subjects). These data are used for the training phase. The mask detector studies these images to learn the thoracic characteristics.
- COVID-19-B: The public dataset of Ma et al. [62], which consists of annotated volumes of CT images (COVID-19), for network training. The scans were obtained via the Coronacases initiative and the Radiopaedia database.
- COVID-19-C: publicly available database with thoracic CT images [63]. (These data are used for the test phase.)
- COVID-19-D: A collection of 3D CT images of 10 confirmed COVID-19 cases, which are made available online by the Coronacases initiative [64]. (These data are used for the test phase).

The primary goal of data selection was to make the data accessible so that academics could access and enhance them. The utilization of these datasets in future research may potentially allow for a more accurate diagnosis of COVID-19 patients.

### 4.2. Evaluation Metrics

We utilized the most-used assessment measures to assess the performance of the proposed approaches, i.e., accuracy, precision, Dice coefficient, the area under the curve, sensitivity, specificity, and F1 score, which are calculated as illustrated in Table 2:

To compute accuracy, sensitivity, specificity, precision, and F1, we used the confusion matrix for the definitions of true positive (TP), false positive (FP), true negative (TN), and false negative (FN).

### 4.3. Evaluation of the DRL System

**Figure 7:**
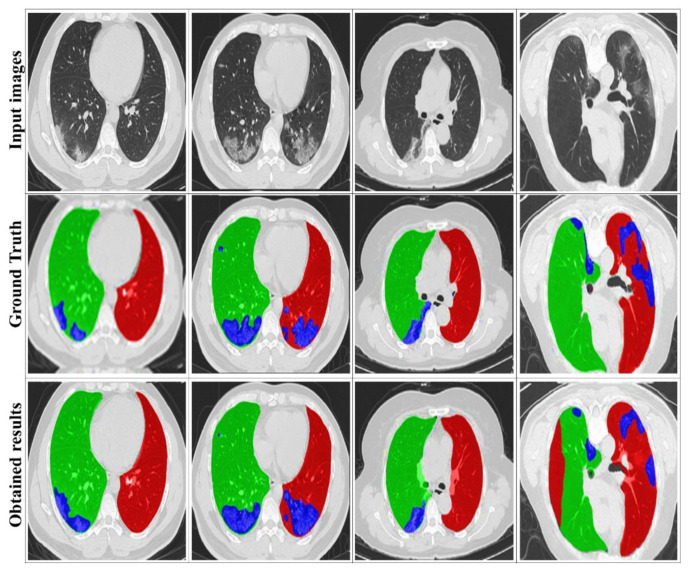
Examples of visualized segmentation results. The red, green, and blue colors respectively denote the left lung, the right lung, and the infection.

Figure 7 illustrates some examples of the visualized segmentation outcomes. It can be found that the suggested approach yields decent results, particularly for the two lung sides, due to the extracted masks from training datasets. This smooths the segmentation of the infection area, thanks to DRL segmentation.

The RLd subset is used to obtain the rewards for the DQN and for the selection of the hyperparameters, which are chosen based on the best configuration for the two baselines and our approach. The mean and standard deviation of the five separate runs are reported (five random seeds). The DQN network is trained using stochastic gradient descent (SGD) with momentum, weight decay set to 103, and a training batch size of 16.

The network is trained using the *TLd* set with a small fixed budget of 0.5k regions to encourage the selection of regions that will improve learning performance. For different budgets, we assess the learned acquisition function as well as the baselines on EvLs, where we request labels until the budget is met. Once the budget is met, a synthetic dataset including a substantial amount of labeled data is generated without the need for human intervention. We assess the final segmentation′s performance (as assessed by average IoU) on the COVID-19-B test set, and we validate it using the COVID-19-C set.

Our results are compared to three distinct points:

1. Ea is the uniform random selection of areas to be labeled from all potential regions in the dataset at each phase;
2. Ei  is an uncertainty sampling approach that picks voxel-level areas with the highest cumulative Shannon entropy;
3. Eb chooses locations with the highest cumulative BALD measure at the pixel level [65].

In the earliest studies, we utilized 20 iterations for computational reasons. We saw no improvement after more than 20 repetitions.

**Figure 8:**
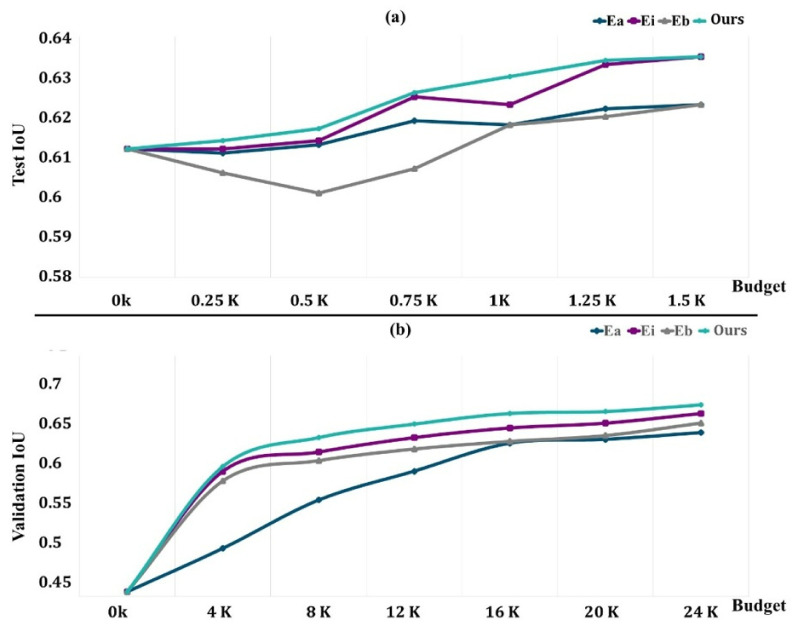
Performance evaluation of methods with an increasingly active learning budget ((**a**) the test performance variations; (**b**) the validation performance variations).

Figure 8a depicts the findings on COVID-19-B for various budget amounts. Except for the 1.5k areas, where we obtain performance comparable to that of Ei, our technique outperforms the baseline performance for any given budget. We believe that because the dataset contains a limited number of pictures, selecting 1.5k areas already achieves more than 98 percent of the maximum performance, and the differences between our technique and Ei are trivial. Surprisingly, Eb is worse than Ea, particularly for low-budget situations when training with newly obtained labels gives no further details. It rapidly adjusts to the training, producing a worse outcome than with the first weights.

The findings for the various budgets are shown in Figure 8b. In this case, we can also see that our strategy outperforms all other budget points. We acquired a performance of 64.5% of the average unit of labor value by labeling sections of 20,000 pixels, which amount to just 6% of the total pixels (in addition to the labeled data in *TLd*). This was 96% of the segmentation network’s performance if it had access to all labeled pixels. Ei requires 6k more named areas to obtain the same performance (about 30% more pixels, which is equivalent to 45 more images). In this bigger dataset, Eb outperforms random, indicating that for the segmentation job, Eb may only begin to show its benefits with substantially larger budgets.

**Figure 9:**
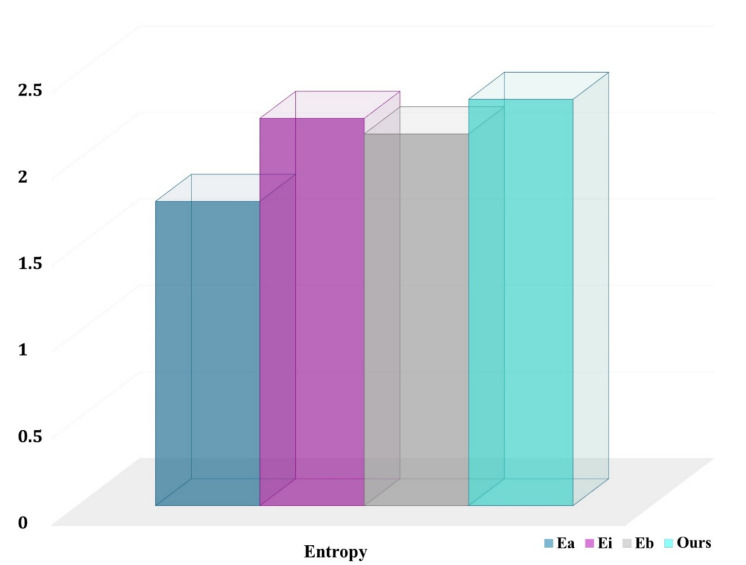
Entropy of class distributions obtained from voxels of selected regions.

Our technique is especially effective for underrepresented classes such as normal, COVID-19, and pneumonia. In fact, our technique chooses more pixels from underrepresented groups than the baselines. This is an unintended consequence of explicitly optimizing for the average job level and developing representations of the class-aware for actions and states. The entropy for chosen pixel distribution in the final labeled set (with a budget of 12,000 areas) for a randomly selected COVID-19-C 3D picture is shown in Figure 9. The higher the entropy, the closer the distribution is to being uniform across classes, and our technique has the maximum entropy.

We suggested a data and region-based active learning strategy for 3D semantic segmentation, which is implemented by using reinforcement learning and aims to reduce the time-consuming process of label generation. We presented a new DQN formulation modification for acquisition function learning that is appropriate to the semantic segmentation of large-scale medical pictures. This yields a computationally efficient method that employs less labeled data than the relatively uncommon baselines in the medical imaging sector while attaining the same performance. Furthermore, because our technique directly optimizes for the average IoU per class and defines class-based representations of states and actions, it necessitates more labels from underrepresented classes than baselines. This enhances performance and helps to reduce the class imbalance.

### 4.4. Comparison with State-of-the-Art Methods

**Figure 10:**
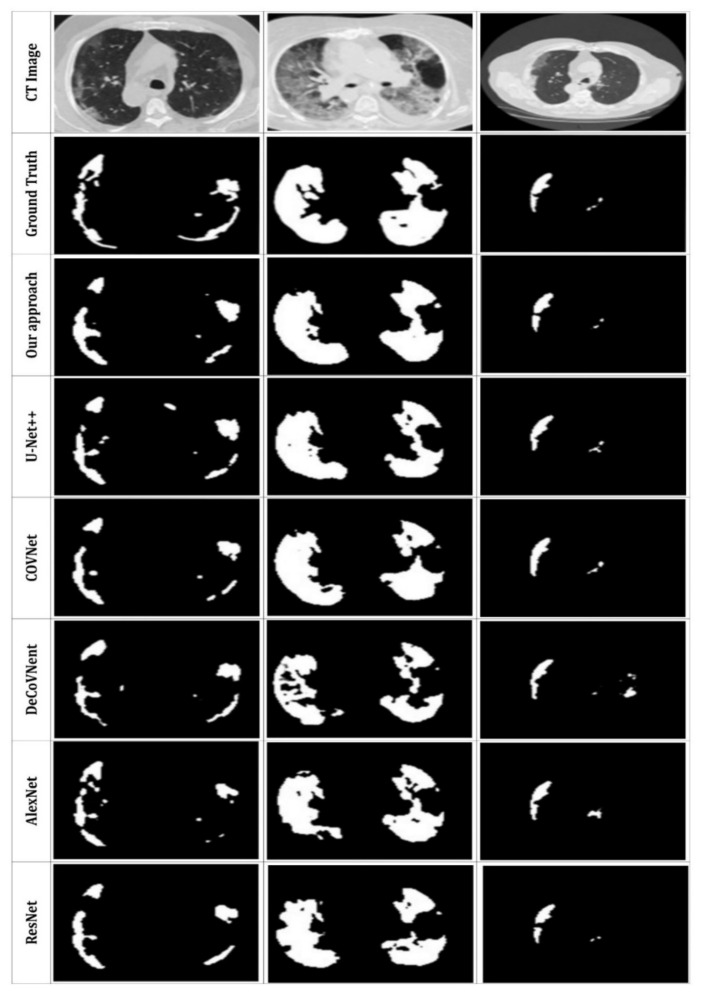
Visual comparison of the segmentation results with other, former models.

The quantitative findings for the suggested system are presented in this subsection; Figure 10 and Table 3 compare the qualitative and quantitative results to the state-of-the-art used techniques. To demonstrate the performance of the suggested DRL model, we tested various former approaches (U-Net++ [43], COVNet [42], DeCoVNet [39], AlexNet [20], ResNet [18]) using the same CT datasets.

Based on Figure 10, the suggested method can properly segment the lung CT images and detect the infected areas, specifically the minor ones. In contrast, the U-Net++ network presents more over-segmented regions. Further COVNet, DeCoVNet, AlexNet and ResNet present good segmentation results but with less definite for smaller segments. Our segmentation results are the closest to the ground truth. This proves the efficiency of the proposed approach. Furthermore, we can also see that the use of MAS to extract the image masks can accurately enhance the segmentation and allow the detection of complicated infection areas in CT scans, which indicates the efficacy of the strategy described in this study.

Table 3 depicts the achieved performance rates. It can be found that the segmentation results for COVID-19 infection have relatively high accuracy as well as a good Dice coefficient, indicating that the proposed model affords better results in the segmentation pipeline. Additionally, using MAS in place of traditional mask extraction ensures global feature detection, encourages feature learning and reuse, and significantly improves the segmentation outcomes.

In this study, we used an advanced DRL system to assess COVID-19, which represents a serious factor in the status of severe pneumonia. Furthermore, most of the previous studies showed competitive results. On the basis of our experimental investigations, we believe that the use of DRL has a significant impact on obtaining medical image information for larger lesions or objects. Coronavirus infection has surprised all populations by its rapid spread and has had a major impact on the lives of billions of people. Chest examinations have been shown to be an effective tool for the detection, quantification of damaged areas, and monitoring of the disease. Deep learning algorithms can be developed to assist in the analysis of a potentially large number of 3D thoracic images to support the efforts of medical teams and point out areas not observable during a simple 3D image check. The biggest constraint, however, is the limited number of COVID-19 CT scans. The current research focused on how MAS can learn from limited training instances. As a result, we feel that the number of training datasets is enough for our DRL technique. More importantly, the present research may be applied to common mask extraction difficulties. Furthermore, the number of test cases is equivalent to current COVID-19 segmentation studies. Another limitation is that the innovative contribution of DRL seems to be limited due to MAS interactions. However, this was not the main goal of this study. Rather, our primary goal is to establish a solid foundation for future work in deep reinforcement learning with limited datasets, and we believe that the data, as well as the process described in this study, could attract more attention in the field.

Our experiments demonstrated that our approach significantly surpasses the state-of-the-art on a benchmark dataset for image segmentation, particularly when noisy and conflicting pairwise labels are used in training. Reinforcement learning experiments showed that MAS is an effective system for small dataset situations and that the convincingness of agent cooperation can be predicted fairly even with only a small number of samples. The results also showed that image objects provide complementary information and that the proposed method can be used to automatically identify relevant features.

## 5. Conclusions and Future Works

The current AI vision systems, dedicated to medical uses, have proven their strong capability for automatic detection or prediction of diseases and have become a crucial pillar in medical and scientific work, responding to the evolving trend in world population needs. Among the puzzles that have drawn the attention of the world, the pandemic disease coronavirus 2019 (COVID-19) causes huge changes in the human respiratory system, which can be visualized through 3D images. In this research, we provided a robust strategy for semantic segmentation that uses a deep reinforcement learning model. The working principle has been tested several times on different types of medical images. The goal was to be able to address one of the conundrums that is attracting the attention of the current scientific community. With deep reinforcement learning, we have proposed a unique way to minimize human effort in the extraction of medical image masks. We introduced an advanced version of the DQN architecture. As such, our approach enhances deep reinforcement learning to select optimal masks during the segmentation of medical images. The mask extraction stage could be improved in the future. In addition, more complex algorithms, approaches, and datasets appear promising. Deep reinforcement learning may be expanded to provide more possibilities and improve system performance.

## Figures and Tables

**Table 1:** Statistical description of evaluation datasets.

DataSet	%Slice with Infection
COVID-19-A [61]	_
COVID-19-B [62]	100%
COVID-19-C [63]	44.9%
COVID-19-D [64]	52.3%

**Table 2:** Summary of adopted evaluation metrics.

Metrics	Formulas	Description
Accuracy (ACC)	$\frac{\mathrm{TP}+\mathrm{TN}}{\mathrm{TP}+\mathrm{TN}+\;\mathrm{FP}+\mathrm{FN}}$	The ratio of correctly predicted pixels to the total number of pixels in the processed image.
Precision (P_c_)	$\frac{\mathrm{TP}}{\mathrm{TP}+\mathrm{FP}}$	The ratio of correctly predicted lesion pixels to the total of expected lesion pixels.
Sensitivity (Sen)	$\frac{\mathrm{TP}}{\mathrm{TP}+\mathrm{FN}}$	The ratio of the correctly predicted lesion pixels to the total number of real lesion pixels.
F1 score (F1)	$2.\frac{\mathrm{Precision}\;.\;\mathrm{Recall}}{\mathrm{Precision}+\mathrm{Recall}}$	The ratio obtained from a combination of both precision and sensitivity results.
Specificity (S_p_)	$\frac{\mathrm{TN}}{\mathrm{TN}+\mathrm{FP}}$	The ratio of correctly predicted normal pixels to the total number of actual normal pixels.
Dice coefficient (DC)	$\frac{2.\left(X\cap Y\right)}{X+Y}$	The similarity between the method output (Y) and the ground truth (X).
Structural metric (S_m_)	Sm = (1 − β).Sos(Sop,Sgt) + β.Sor(Sop,Sgt)	The structural similarity between the prediction map and ground truth mask.
Mean Absolute Error (MAE)	$\mathrm{MAE}=\frac{1}{w\;.\;h}$ ∑wi∑hj|Sop(i,j) − Sgt(i,j)|	Measures the pixel-wise difference.

**Table 3:** Quantitative comparisons of ground truth for different, former models.

	ACC	DC	Sen	Sp	Pc	F1	Sm	MAE
Our approach	0.9712	0.8081	0.7997	0.9948	0.8621	0.8301	0.8438	0.0086
U-Net++ [43]	0.9687	0.7972	0.7845	0.9952	0.8437	0.8206	0.8623	0.0085
COVNet [42]	0.9698	0.7754	0.7400	0.9959	0.8470	0.7930	0.8334	0.0094
DeCoVNet [39]	0.9697	0.8020	0.8106	0.9962	0.8347	0.8116	0.8511	0.0107
AlexNet [20]	0.8900	0.6910	0.8110	0.9930	0.9500	0.8062	0.8475	0.0125
ResNet [18]	0.8984	0.7408	0.7608	0.9937	0.7549	0.7558	0.8080	0.0157

## Data Availability

Not applicable.
